# The post-translational adaptor protein SadB modulates the pathogenicity of *Pseudomonas aeruginosa*

**DOI:** 10.1128/jb.00437-25

**Published:** 2026-03-24

**Authors:** Maria Papangeli, Jeni Luckett, Stephan Heeb, Morgan R. Alexander, Paul Williams, Jean-Frédéric Dubern

**Affiliations:** 1 National Biofilm Innovation Centre, Biodiscovery Institute & School of Life Sciences, University of Nottingham 105592, Nottingham, United Kingdom; 2 Advanced Materials and Healthcare Technologies, School of Pharmacy, University of Nottingham 14302, Nottingham, United Kingdom; Dartmouth College Geisel School of Medicine, Hanover, New Hampshire, USA

**Keywords:** *Pseudomonas aeruginosa*, SadB, virulence, infection model, biofilms, swarming, transcriptome, rhamnolipids, signalling, quorum sensing

## Abstract

In *Pseudomonas aeruginosa,* SadB acts as a post-translational adaptor protein that binds to the transcriptional regulator AmrZ. Deletion of *sadB* results in a biofilm-defective, hyperswarming phenotype. To investigate whether SadB contributes to virulence, we employed *in vivo* bioluminescence imaging and histopathology to visualize the development of infection in a mouse soft tissue model. Compared with the parent PAO1 strain, the *sadB* mutant was highly attenuated and rapidly cleared from the infection site, whereas genetic complementation conferring constitutive expression of *sadB* resulted in a much more persistent phenotype. Transcriptome analysis was undertaken to gain insights into the global impact of SadB, revealing that it modulates expression of diverse genes involved in biofilm development, quorum sensing (QS), secondary metabolite production, iron acquisition, virulence, protein secretion, and anaerobiosis. In the *sadB* mutant*,* we observed log-phase induction of the *rhl* and *pqs* QS systems, increased production of siderophores and pyocyanin, differential regulation of genes involved in c-di-GMP signaling, and a growth defect under microaerophilic conditions. The overproduction of rhamnolipids is consistent with the hyperswarming, biofilm-defective phenotype of the *sadB* mutant since rhamnolipids act as anti-adhesive surface lubricants. Deletion of *rhlA* in the *sadB* mutant resulted in the restoration of biofilm formation, offering mechanistic insight into the biofilm-defective phenotype of the *sadB* mutant. SadB clearly plays a global role in the adaptive behavior and virulence of *P. aeruginosa*.

**IMPORTANCE:**

Biofilms are characterized by their intrinsic tolerance to antibiotics, host immune defenses, and ability to cause persistent infections. In *Pseudomonas aeruginosa,* mutation of the surface attachment defect gene*, sadB,* results in cells that are biofilm-defective, hyperswarmers. Here, we sought to determine whether SadB regulates virulence and influences the development of infection. In a mouse skin infection model, a *P. aeruginosa sadB* deletion mutant was highly attenuated. We also demonstrate that SadB regulates many different genes involved in virulence, quorum sensing, iron acquisition, protein secretion, and anaerobiosis as well as biofilm formation, highlighting a broader role in pathogenesis than previously recognized. Consequently, SadB has potential as a novel protein target for antibacterial drug discovery.

## INTRODUCTION

*Pseudomonas aeruginosa* is an opportunistic human pathogen that produces an armory of cell-associated and extracellular virulence determinants controlled via global regulatory systems operating at both transcriptional and post-transcriptional levels (1). It can adopt either planktonic free-living or sessile biofilm-associated lifestyles, which respectively contribute to acute and chronic infections. *P. aeruginosa* is capable of flagella-mediated swimming through liquids or attaching and migrating over surfaces via swarming or type IV pilus-mediated twitching motility (2). Both flagella and type IV pili can also act as adhesins and mechanosensors facilitating biotic and abiotic surface colonization (3). With respect to biofilm development, the first committed step involves the transition from reversible (attachment via the cell pole) to irreversible surface attachment (via the long axis of the cell) (2, 4). This, in turn, leads to micro- and macro-colony formation and biofilm maturation, during which the bacteria become embedded within an extracellular matrix consisting of exopolysaccharides, extracellular DNA, lipids, and proteins (5–7).

Biofilm formation and swarming motility in *P. aeruginosa* are inversely regulated. One of the first *sad* (surface attachment defect) genes discovered to play a role in this regulatory pathway was *sadB*. A transposon insertion in the *P. aeruginosa* strain PA14 *sadB* gene enhanced swarming but prevented biofilm formation (8, 9). Conversely, overexpression of *sadB* promoted biofilm development but reduced swarming motility. Further work resulted in a model for *P. aeruginosa* PA14 in which the SadB-dependent inverse regulation of swarming and biofilm was proposed to be mediated via viscosity-dependent modulation of flagellar reversal rates and to influence Pel exopolysaccharide production via a mechanism involving the *pil-chp* chemotaxis pathway (9, 10).

In diverse bacterial species, the transition between motile and sessile lifestyles is controlled via pathways involving the second messenger cyclic-di-guanylate (c-di-GMP) whereby elevated c-di-GMP levels promote biofilm formation but inhibit motility (2, 11). C-di-GMP signaling depends on the production and turnover of this second messenger via diguanylate cyclases (DGCs) and phosphodiesterases (PDEs), respectively, while cellular responses are mediated via specific c-di-GMP binding receptors (12). High levels of c-di-GMP increase production of key *P. aeruginosa* biofilm components including Pel, Psl, and alginate exopolysaccharides via transcriptional and post-transcriptional regulatory pathways. In *P. aeruginosa* PA14, deletion of the cyclase gene, *sadC,* resulted in a similar biofilm-defective, hyper-swarming phenotype to that of a *sadB* mutant, which could be restored to the WT phenotype by *sadB* overexpression, suggesting that *sadB* acts downstream of *sadC* (13). C-di-GMP produced via SadC is degraded via the PDE, BifA, which together with SadC, inversely regulates biofilm and swarming motility in a *sadB*-dependent manner in *P. aeruginosa* PA14. BifA is considered to act upstream of *sadB* (14), which is highly conserved amongst the pseudomonads but is absent from most other bacterial species. *sadB* codes for a cytoplasmic protein (9) which, in *Pseudomonas fluorescens* F113, has been reported to bind c-di-GMP (15). However, more recently, Ben-David et al. (16) reported that SadB from *P. aeruginosa* does not bind c-di-GMP but instead functions as an adaptor protein, post-translationally modulating the activity of AmrZ, a member of the ribbon-helix-helix family of DNA binding proteins that acts as a transcriptional activator and repressor of multiple genes involved in virulence and biofilm formation (17, 18). SadB binds to the C-terminal domain of AmrZ and promotes the rapid proteolytic degradation of AmrZ primarily via the Lon protease (16).

Here, we confirm that a *P. aeruginosa* PAO1 *sadB* deletion mutant exhibiting the same biofilm-defective, hyper-swarming phenotype observed in strain PA14 is highly attenuated in a mouse infection model and acts as a pleiotropic regulator of diverse genes involved in virulence, quorum sensing, secondary metabolite production, iron acquisition, protein secretion, and anaerobiosis. Biofilm development by Δ*sadB* could be restored by deleting *rhlA,* consistent with the overproduction of rhamnolipids observed in the *sadB* mutant, thus contributing to our understanding of the inability of *P. aeruginosa sadB* mutants to form mature biofilms.

## RESULTS

### A *P. aeruginosa* PAO1 Δ*sadB* mutant is highly attenuated in a mouse wound infection model

**Fig 1:**
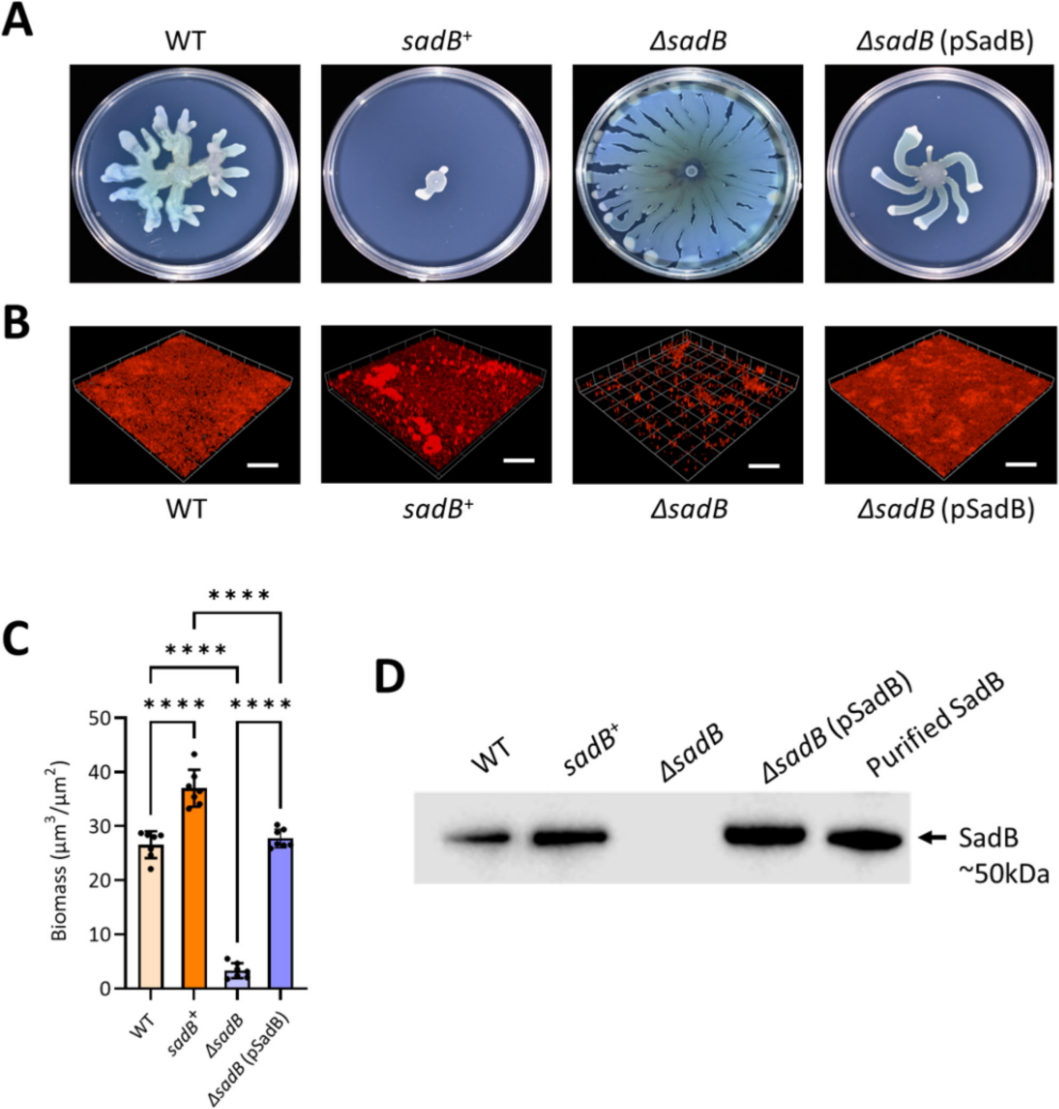
Swarming and biofilm phenotypes of *P. aeruginosa* strains lacking or constitutively overexpressing *sadB*. (**A**) Representative images of swarming motilities displayed by *P. aeruginosa* PAO1 WT compared with *sadB*^+^ (WT transformed with pSadB), Δ*sadB,* and Δ*sadB* genetically complemented with pSadB. (**B**) Confocal images of biofilm formation by the same strains as in panel **A** after 48 h of incubation in RPMI-1640 medium. Biofilms were stained with FM4-64 dye. Scale bar: 100 µm. (**C**) Quantification of biofilm biomass from confocal images shown in panel **B**. Statistical differences between group means were determined by one-way ANOVA using Tukey’s multiple comparisons test (****P < 0.0001). (**D**) Detection of SadB in cytoplasmic extracts of the *P. aeruginosa* strains as in panel **A** by immunoblotting compared with the purified recombinant SadB protein.

We first confirmed that deletion of *sadB* had a similar impact on swarming and biofilm formation in *P. aeruginosa* as that described by others (10, 16, 19) (see Table S1 at https://doi.org/10.5281/zenodo.19057088). The PAO1 Δ*sadB* strain constructed was a hyper-swarmer (Fig. 1A), failed to form a mature biofilm (Fig. 1B and C; see Fig. S4A and B at https://doi.org/10.5281/zenodo.19057088), and did not express *sadB* (Fig. 1D). Genetic complementation was achieved by introducing *sadB* on a plasmid expression vector (Fig. 1D; see Table S1 at https://doi.org/10.5281/zenodo.19057088). To evaluate the contribution of SadB to colonization and persistence *in vivo,* we used a simple and reproducible mouse model that closely mimics human cutaneous wound infections without requiring high bacterial inocula or traumatic injury (20) and that could be followed non-invasively using *in vivo* bioluminescence imaging (21–23). This facilitates quantitative real-time imaging for monitoring the progress of infection, in which bioluminescence intensity correlates with bacterial load (22, 24). For these experiments, we constructed bioluminescent *P. aeruginosa* PAO1 wild type (WT) and a Δ*sadB* mutant by introducing a CTX::*tac’-luxCDABE* fusion onto the chromosome to avoid the loss during infection that accompanies the use of plasmid expression vectors. In addition, we chromosomally complemented the Δ*sadB* mutant with a CTX::*tac’-sadB-luxCDABE* fusion that constitutively expresses *sadB* (see Table S1 at https://doi.org/10.5281/zenodo.19057088). The phenotype of each bioluminescent strain with respect to *sadB* expression and swarming was confirmed (see Fig. S1 at https://doi.org/10.5281/zenodo.19057088).

**Fig 2:**
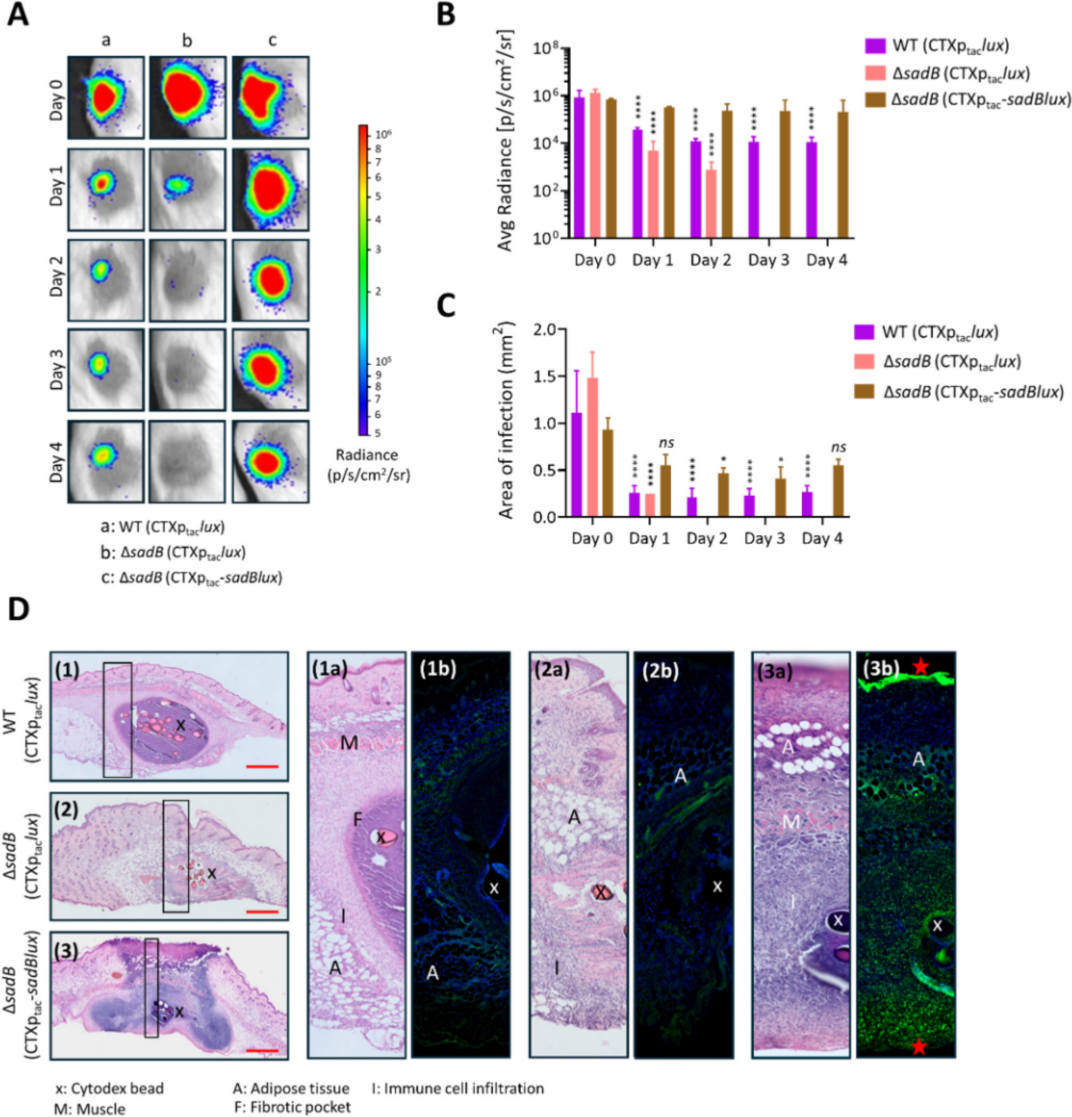
*P. aeruginosa* Δs*adB* is highly attenuated in a mouse subcutaneous infection model compared with WT and *sadB*^+^. (**A**) Luminescent images of the infection sites in live mice over 4 days after inoculation with bioluminescent *P. aeruginosa* strains (WT [CTX::*tac’-luxCDABE*], Δ*sadB* [CTX::*tac’-luxCDABE*], and Δ*sadB* [CTX::*tac’-sadB-luxCDABE*]). *n* = 4 mice per bacterial genotype were imaged using an IVIS Spectrum after inoculation (day 0) and on each subsequent day for 4 days. (**B**) Quantification of metabolically active bacteria and (**C**) quantification of the area of infection for each strain imaged. Statistical differences between group means were determined by two-way ANOVA using Tukey’s multiple comparisons test to day 0 (*P < 0.05, ****P < 0.0001). (**D**) Histological assessment of *P. aeruginosa* infection site tissue. Panels 1, 2, and 3 show hematoxylin and eosin staining of excised tissues from mice infected respectively with *P. aeruginosa* WT*,* Δ*sadB,* and Δ*sadB* (CTX::*tac’-sadB-luxCDABE*). The boxed regions in each panel are shown as enlarged panels in 1a, 2a, and 3a. *P. aeruginosa* WT infection established within a fibrotic pocket (panels 1 and 1a), whereas no defined fibrotic pockets were apparent following Δ*sadB* (panels 2 and 2a) or Δ*sadB* (CTX::*tac’-sadB-luxCDABE*) (panels 3 and 3a). For panels 1b, 2b, and 3b, bacterial cells were visualized by IHC using polyclonal antibodies raised against *P. aeruginosa* (green) and tissue sections counterstained for DNA with POPO-1 (blue). Panel 3b shows that for Δ*sadB* (CTX::*tac’-sadB-luxCDABE*)*,* bacterial cells were present on the dermal surface and in clusters at the interface between the mouse skin and the underlying tissue (see red stars). In addition, although no fibrotic pocket formed, there is evidence of leukocyte infiltration in panel 3b. A, adipose tissue; M, muscle; F, fibrotic pocket; I, immune cell infiltration; Cytodex beads (x).

Each of the three *P. aeruginosa* strains (1 × 10^5^ cells) was mixed with Cytodex 1 beads and injected subcutaneously into mice. The progress of infection was tracked by imaging the bioluminescent bacteria each day for 4 days. Figure 2 shows that the WT established within the dermis inoculation site, whereas the Δ*sadB* mutant was unable to colonize. Compared with the parent strain, no bioluminescence was detectable in 3/4 mice, suggesting that in these, no live Δ*sadB* cells were present after 1 day post-infection (Fig. 2A through C). Furthermore, no viable bacteria could be isolated from the Δ*sadB* infection by viable counting (see Fig. S2 at https://doi.org/10.5281/zenodo.19057088). Genetic complementation of the Δ*sadB* mutant, in which *sadB* expression was driven via a constitutive p*tac* promoter, restored the ability of *P. aeruginosa* to establish infection and to colonize a much larger infection site than the parent WT strain (Fig. 2A through D).

**Fig 3:**
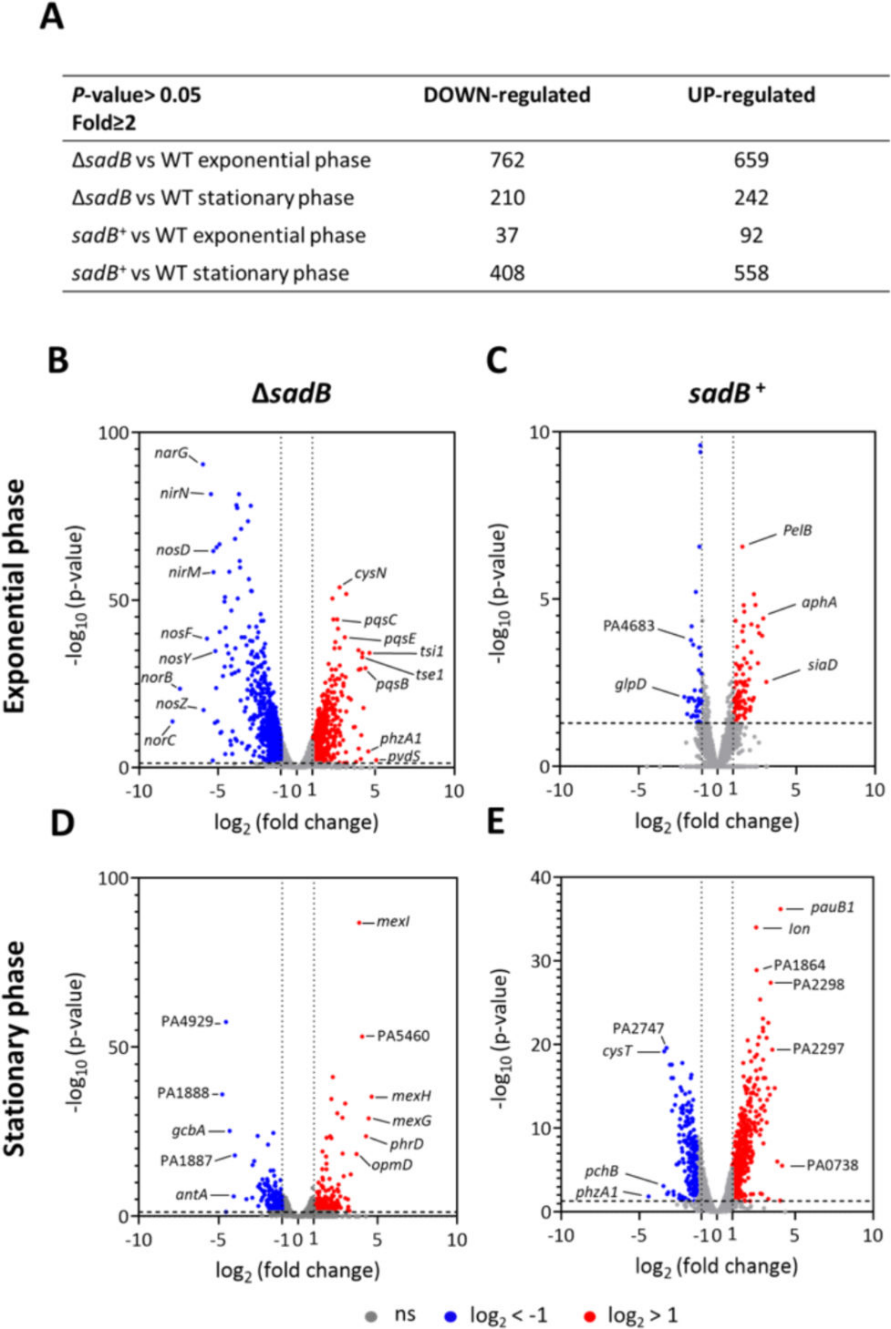
Comparison of the transcriptomes of *P. aeruginosa* Δ*sadB* and *sadB^+^* with WT after growth in LB (5 mL in 50 mL conical tubes) with shaking at 37°C from cells harvested at exponential (OD_600_ 0.7) and stationary (OD_600_ ~2.0) phases, respectively. (**A**) Total number of genes up- and downregulated. (**B–E**) Volcano plots of the differentially expressed Δ*sadB* (**B, D**) and *sadB^+^* (**C, E**) genes compared with the WT during exponential (**B, C**) and stationary phases (**D, E**) of growth. Upregulated genes are indicated in red (log_2_ fold change > 1) and downregulated genes in blue (log_2_ fold change < −1), while gray indicates no significant changes. Statistical cut-off: *P*-value <0.05.

On day 4, the mice were euthanized and the intact infection sites dissected, formalin-fixed, and embedded in wax. The architecture and spatial localization of the infection site was assessed by comparing parallel tissue sections of each site, one stained with hematoxylin and eosin and the other used for immunohistochemistry (IHC) to localize the *P. aeruginosa* cells in each infection site. Histopathological assessment of the dermis infection site revealed marked strain-dependent differences in tissue architecture (Fig. 2D). The WT underwent encapsulation within a discrete fibrotic pocket (into which leukocyte infiltration was observed). In contrast, for Δ*sadB*, no fibrotic pocket formed, although some leukocyte infiltration was visible. Inoculation with the Δ*sadB* mutant constitutively expressing *sadB* did not lead to the formation of fibrotic pockets and stimulated marked infiltration of leukocytes within and beyond the infection site into the adipose and subdermal tissue layers. IHC was used to localize *P. aeruginosa* WT, Δ*sadB,* and Δ*sadB* CTX::*tac’-sadB-luxCDABE* cells within, as well as outside, the excised and sectioned infection sites. These images [Fig. 2D, (1b), (2b), and (3b)] revealed the presence of WT bacteria both inside and outside the fibrotic pocket within the infection site [Fig. 2D (1b)]. A few Δ*sadB* cells were only detected within the infection site [Fig. 2D (2b)] while Δ*sadB* CTX::*tac’-sadB-luxCDABE* cells were observed within and outside the infection site, including colonization of the top of the dermal surface [Fig. 2D (3b)].

### Transcriptomic analysis reveals a highly pleiotropic regulatory role for SadB

To determine the transcriptional impact of deleting or constitutively expressing *sadB*, RNA-seq was performed on total RNA extracted, respectively, from the WT, Δ*sadB,* and *sadB*^+^ (i.e., WT [pSadB]; see Table S1 at https://doi.org/10.5281/zenodo.19057088) strains cultured planktonically with shaking at 37°C in lysogeny broth (LB) and harvested at both mid-exponential (OD_600_ 0.7) and stationary phase (OD_600_ ~2). Following statistical validation of the data set obtained, only genes with a fold change ≥ ±2 and an adjusted *P*-value of ≤0.05 were considered. Analysis of differentially regulated genes revealed that a significant proportion of the transcriptome was affected (Fig. 3). Comparison of Δ*sadB* with the WT grown to exponential phase revealed differential regulation of ~24% of the genome (659 upregulated genes; 762 downregulated genes; Fig. 3). In the stationary phase, ~8% were differentially regulated (242 upregulated genes; 210 downregulated genes). Constitutive expression of *sadB* in the *sadB*^+^ strain compared with the parent resulted in differential expression of ~2% of the genome in exponential phase (92 upregulated genes; 37 downregulated genes). However, in the stationary phase, ~17% of the genome was differentially regulated (558 upregulated genes; 408 downregulated genes) (Fig. 3). Of the 648 genes upregulated in Δ*sadB* in log phase, only 20 remained upregulated in stationary phase. Of the 762 genes downregulated in Δ*sadB* in log phase, only 210 remained downregulated in stationary phase (Fig. 3), suggesting a major regulatory role for SadB in planktonic culture during the exponential phase of growth. The key differentially regulated genes are summarized in Table S2 at https://doi.org/10.5281/zenodo.19057088.

As anticipated, many of the genes at least twofold differentially downregulated in the Δ*sadB* mutant are involved in biofilm development (see Table S2 at https://doi.org/10.5281/zenodo.19057088). These include those coding for c-di-GMP generation (e.g., the DGCs, SadC, and GbcA), c-di-GMP receptors (e.g., *pilZ*), flagella (e.g., *fliC, motB,* and *flgM*), pili (e.g., *pilA* and *cupA1*), and exopolysaccharides (e.g., *pslA*). Overall, the RNA-seq data revealed that diverse genes involved in virulence, protein secretion (type II, III, and VI systems), iron and heme acquisition and storage, transcriptional and post-transcriptional gene regulation, quorum sensing (QS), primary and secondary metabolism, and respiration were differentially regulated via SadB during log-phase planktonic growth when compared with WT (see Table S2 at https://doi.org/10.5281/zenodo.19057088). The most downregulated genes in log-phase Δ*sadB* were those involved in denitrification (*nar, nir, nor,* and *nos*) (Fig. 3B; see Table S2 at https://doi.org/10.5281/zenodo.19057088).

**Fig 4:**
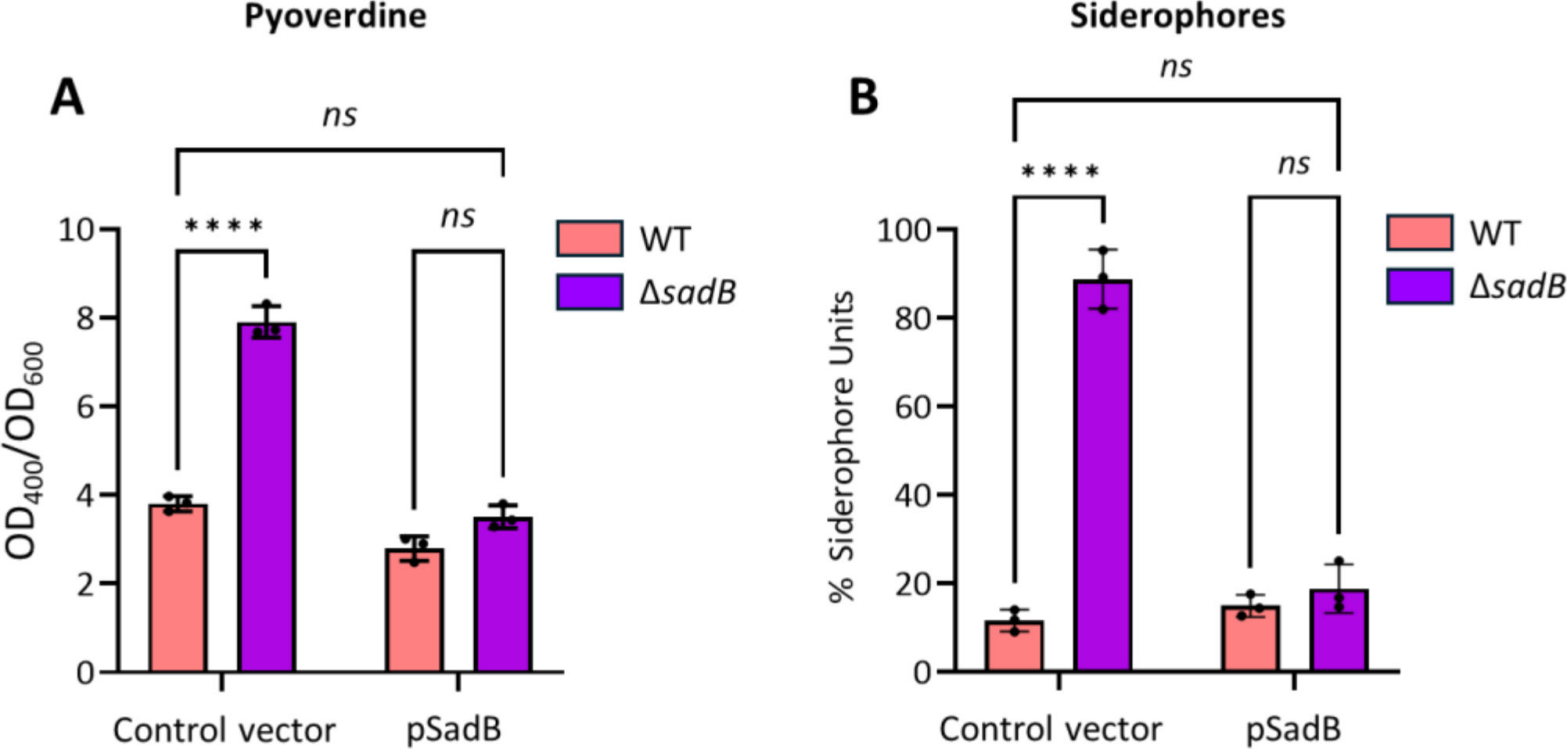
SadB negatively regulates siderophore production. (**A**) Pyoverdine production quantified by determination of the OD_400_ in cell-free spent culture supernatants of the WT and Δ*sadB* mutant strains harboring the control vector pME6032Δ*lacIQ* or pSadB, grown in CAA medium at 37°C for 24 h. (**B**) Total siderophore production measured using the CAS method from the supernatants of the four strains tested, grown in CAA medium at 37°C for 24 h. Values given are averages from at least three different cultures ± standard deviation. Statistical differences between group means were determined by two-way ANOVA using Tukey’s multiple comparisons test (****P < 0.0001).

When *P. aeruginosa* is grown in iron-rich conditions, the *pch* and *pvd* genes required for pyochelin and pyoverdine siderophore synthesis and transport, genes coding for outer membrane receptors for exogenous catechol and hydroxamate siderophore uptake, as well as the *has* and *hxu* genes required for heme acquisition, are repressed via Fur to prevent iron toxicity (25, 26). Conversely, Fur positively regulates the genes coding for the bacterioferritin (*bfrB*) and ferritin (*ftnA*) iron storage proteins via negative regulation of the PrrF sRNAs. Consistent with these data, *fur, brfB,* and *ftnA* were downregulated in the Δ*sadB* mutant in log phase while many of the *pch*, *pvd*, *has,* and *hxu* genes as well as *prrF2* were upregulated, suggesting a role for SadB in iron metabolism (see Table S2 at https://doi.org/10.5281/zenodo.19057088). In agreement with the RNA-seq data, deletion of *sadB* resulted in a statistically significant increase in total siderophore production, specifically with respect to pyoverdine (Fig. 4).

The increased expression of the *pqs* genes (Fig. 3B; see Table S2 at https://doi.org/10.5281/zenodo.19057088) observed in Δ*sadB* is in agreement with the PrrF-dependent enhancement of 2-heptyl-3-hydroxy-4-quinolone (PQS) production (27). This occurs through repression of the anthranilate degradation genes (*antABC*) such that anthranilate can be redirected towards synthesis of 2-alkyl-4-quinolones (AQs), including PQS. Downregulation of the *antABC* genes in Δ*sadB* (Fig. 3D; see Table S2 at https://doi.org/10.5281/zenodo.19057088) is therefore consistent with the PrrF-dependent downregulation of anthranilate degradation (27).

In *sadB^+^*, where sadB is constitutively overexpressed in the WT, the most highly upregulated genes in log-phase cells included the biofilm-associated genes *cdrA* and *cdrB*, the *psl* and *pel* exopolysaccharide biosynthesis genes, and the *siaABCD* signaling network (see Table S2 at https://doi.org/10.5281/zenodo.19057088). These genes are also known to be regulated via AmrZ (16, 18). Examples of genes upregulated in Δ*sadB* in log phase but downregulated in *sadB*^+^ in stationary phase included those involved in iron acquisition (e.g., *pch*, *pvd,* and *hxu*) and genes regulated via QS, including *phzA1*, *lasB,* and *hcnABC* (see Table S2 at https://doi.org/10.5281/zenodo.19057088).

When collectively considered, the RNA-seq data obtained suggest that SadB has a highly pleiotropic effect on transcription. Deletion of *sadB* had the greatest impact on log-phase planktonic cells, whereas when constitutively upregulated in *sadB*^+^, many more genes were differentially regulated in the stationary phase compared with log phase (Fig. 3; see Table S2 at https://doi.org/10.5281/zenodo.19057088). Given the recently reported function of SadB as an adaptor protein modulating the function of AmrZ (16), many of the genes differentially regulated via SadB are targets of AmrZ (18). Those that are differentially regulated by AmrZ ≥2-fold are highlighted in Table S2 at https://doi.org/10.5281/zenodo.19057088.

### SadB and c-di-GMP signaling

**Fig 5:**
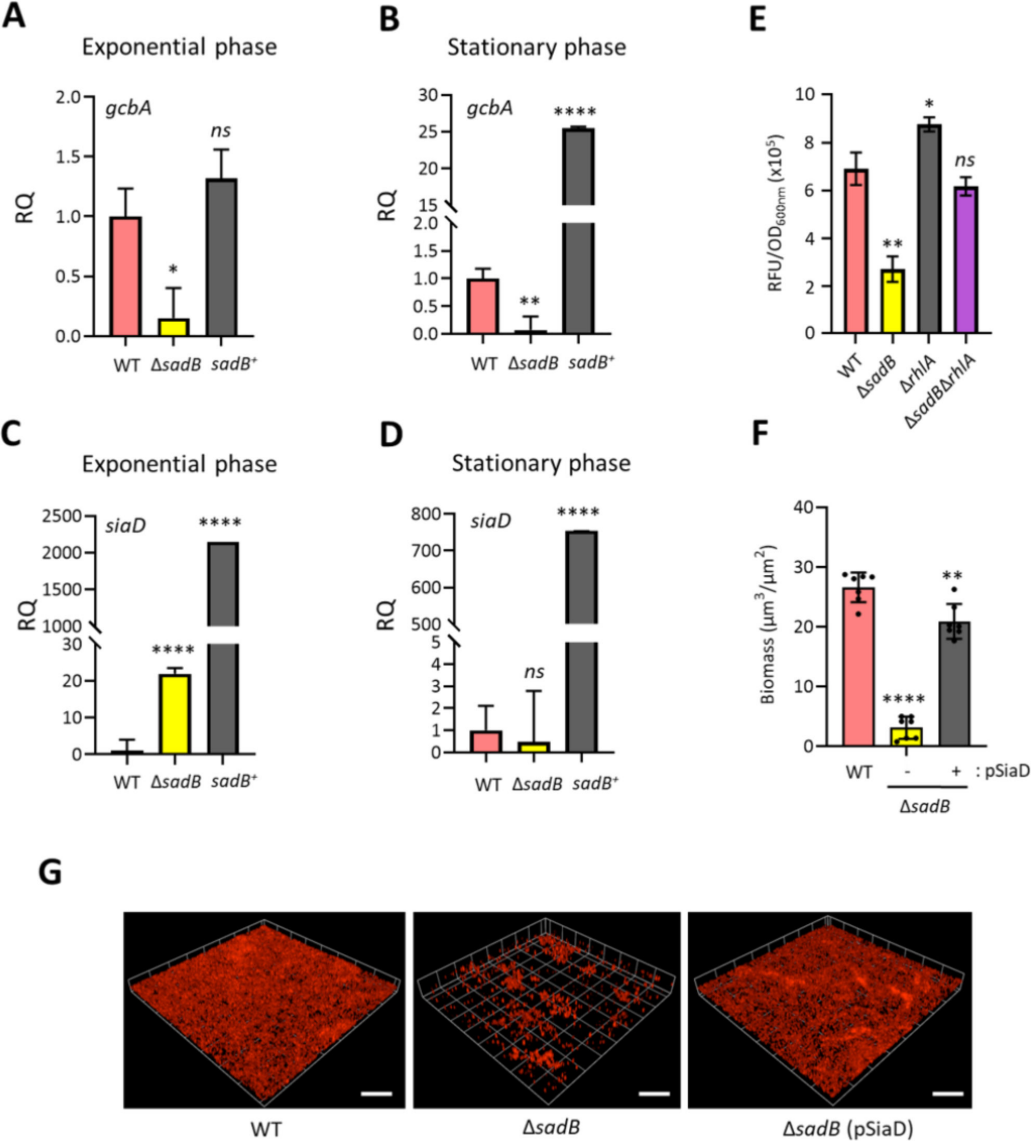
SadB modulates c-di-GMP signaling. Panels **A–D** show that the DGCs, *gcbA* and *siaD,* are differentially expressed in Δ*sadB* and *sadB^+^* compared with WT at both exponential (**A and C**) and stationary (**B and D**) growth phases in LB at 37°C, and quantified by RT-qPCR. (**E**) Indirect quantification of c-di-GMP made using the cdGreen2.1 c-diGMP reporter described by Kaczmarczyk et al. (29). Values correspond to the averages from at least three different cultures ± standard deviation plotted as relative fluorescence units (RFU) normalized to culture density (relative light units [RLU]/OD_600_). (**F**) Quantification of biofilm biomass from confocal images of WT (pME6032Δ*lacIQ*) and Δ*sadB* harboring the control vector pME6032Δ*lacIQ,* and Δ*sadB* (pSiaD). Values given are averages from at least three different cultures ± standard deviation. (**G**) Confocal biofilm images of *P. aeruginosa* WT (pME6032Δ*lacIQ*) and Δ*sadB* with or without pSiaD, stained with FM4-64 dye after 48 h incubation in RPMI-1640 medium. Scale bar: 100 µm. Statistical differences between group means were determined by one-way ANOVA using Tukey’s multiple comparisons test to the WT (*P < 0.05, **P < 0.01, ****P < 0.0001).

RNA-seq analysis revealed that, in log-phase planktonic cells, deletion of *sadB* resulted in the ≥2-fold downregulation of 9/23 genes coding for c-di-GMP DGCs and 5/12 genes that act as c-di-GMP receptors, but with less impact on genes coding for proteins containing EAL or HD-GYP (3/13) domains that act as PDEs (see Table S2 at https://doi.org/10.5281/zenodo.19057088). The most downregulated DGC gene in log-phase Δ*sadB* was *gcbA* (14-fold), which was further reduced in the stationary phase (19.7-fold). In PAO1, the AmrZ-regulated DGC, GcbA (AdcA) (18), is involved in the initial stages of surface attachment and influences flagellum-driven motility by suppressing flagellar reversal rates in a manner independent of viscosity, surface hardness, and exopolysaccharide production (28). The *gcbA* RNA-seq data for *gcbA* were validated using RT-qPCR, which confirmed the downregulation in log phase when Δ*sadB* was compared with the parent strain, but highly upregulated in *sadB*^+^ in stationary phase (Fig. 5A and B).

The SiaABCD signaling network controls cell aggregation and biofilm formation in *P. aeruginosa* and is important for producing c-di-GMP via the AmrZ-regulated DGC, SiaD (18), and cell-associated Psl in planktonic cells (30). While no differential regulation of the *sia* operon was noted in the RNA-seq for Δ*sadB*, the *sia* genes, including *siaD,* were all highly upregulated in log-phase *sadB*^+^ (see Table S2 at https://doi.org/10.5281/zenodo.19057088). Similar results were obtained using RT-qPCR for both log (Fig. 5C) and stationary-phase (Fig. 5D) *siaD*. Figure 5F and G and Fig. S4C and D at https://doi.org/10.5281/zenodo.19057088 show that, when a plasmid-borne copy of *siaD* was introduced into Δ*sadB,* biofilm formation was restored. These data highlighted a role for *sadB* in regulating a subset of genes involved in c-di-GMP production and sensing (see Table S2 at https://doi.org/10.5281/zenodo.19057088).

Ben-David et al. (16) quantified the c-di-GMP in log-phase planktonic *P. aeruginosa* PAO1 and in Δ*sadB* and reported an ~60% reduction in the mutant. We confirmed these data using a reporter fusion constructed by Kaczmarczyk et al. (29) that provides an indirect method for c-di-GMP quantification (Fig. 5E). These data underscore the links between SadB, AmrZ, and c-di-GMP signaling in *P. aeruginosa*.

### SadB and the biofilm extracellular matrix

**Fig 6:**
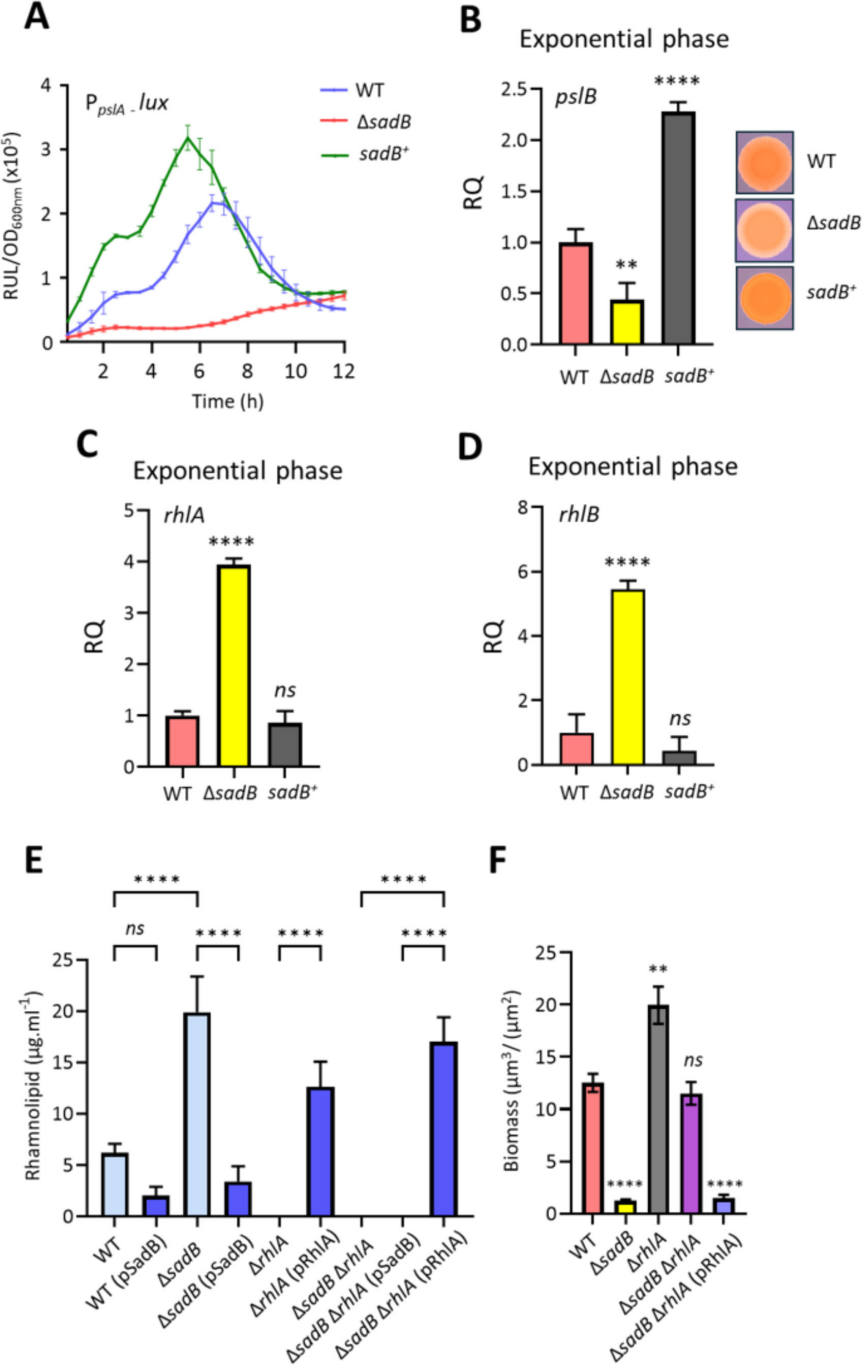
SadB reciprocally regulates genes coding for the exopolysaccharide Psl and the rhamnolipids. (**A**) Transcriptional activity of a *pslA’::lux* promoter fusion in WT and Δ*sadB* mutant with or without pSadB in LB at 37°C. Values correspond to the averages from three different cultures ± standard deviation plotted as relative light units normalized to cell culture density (RLU/OD_600_). Differentially expressed *pslB* (**B**), *rhlA* (**C**), and *rhlB* (**D**) genes of Δ*sadB* mutant and *sadB^+^* overexpressing strains compared to WT during exponential phase of growth at 37°C in LB, quantified using RT-qPCR. Panel B (right-hand side) also shows Congo red staining of WT, Δ*sadB,* and *sadB*^+^ colonies for exopolysaccharide production. (**E**) Rhamnolipid production quantified using LC-MS/MS from the cell-free supernatants prepared from WT, Δ*sadB*, Δ*rhlA*, Δ*sadB*Δ*rhlA,* and the genetically complemented strains after growth in LB at 37°C for 24 h. (**F**) Quantification of biomass from confocal images of WT, Δ*sadB*, Δ*rhlA*, and Δ*sadB*Δ*rhlA* mutants. Values represent the means of at least three biological replicates. Statistical differences between group means were determined by one-way ANOVA using Tukey’s multiple comparisons test to the WT (**P < 0.01, ****P < 0.0001).

Three exopolysaccharides, Psl, Pel, and alginate, are associated with the biofilm matrix in *P. aeruginosa* PAO1. Although alginate is primarily associated with the conversion to mucoidy, there is little alginate present in non-mucoid *P. aeruginosa* PAO1 biofilms (31). RNA-seq revealed that most of the *psl* operon genes were downregulated in log-phase Δ*sadB* but upregulated in the log phase of *sadB*^+^, consistent with the biofilm phenotypes of the respective strains (see Table S2 at https://doi.org/10.5281/zenodo.19057088; Fig. 1B) and the observations of Ben David et al. (16). These data suggest that SadB positively influences *psl* expression and were validated using a CTX::*pslA’-luxCDABE* fusion and RT-qPCR of *pslB* (Fig. 6A and B).

In microtiter plate assays, for both WT and *sadB*^+^ strains, but not Δ*sadB*, *pslA* expression peaked in log phase and was highest in *sadB*^+^ (Fig. 6A). The expression of *pslB* was lowest in Δ*sadB* as quantified using RT-qPCR (Fig. 6B). The reduced Congo red staining of a WT compared with the ΔsadB colonies shown in Fig. 6B is consistent with the reduction in exopolysaccharide production.

Other biofilm-associated and AmrZ target genes that were differentially regulated included the *pel* and *cdrA* operons (upregulated in *sadB*^+^ log-phase cells), the chaperone-usher pilus gene *cupA1* (downregulated in Δ*sadB* but upregulated in *sadB*^+^) and *endA*, an exonuclease involved in eDNA degradation and biofilm dispersal (downregulated in log-phase Δ*sadB*) (see Table S2 at https://doi.org/10.5281/zenodo.19057088; 18).

*P. aeruginosa* produces rhamnolipid surfactants which make multiple contributions to biofilm maturation and architecture ranging from microcolony and channel formation to detachment and dispersal (32–34). Rhamnolipids are also essential for surface swarming migration. Although deletion of *sadB* in strains PA14 (9) and PAO1 (16, 19, and this manuscript) results in a biofilm-defective, hyper-swarming phenotype, the impact of *sadB* on rhamnolipid production has not, to our knowledge, been established.

The expression of *rhlAB* was higher in the Δ*sadB* mutant compared with the WT (*rhlA* 6.8-fold, *rhlB* 1.7-fold) in exponential phase (see Table S2 at https://doi.org/10.5281/zenodo.19057088). RT-qPCR data validated the increased expression of *rhlA* and *rhlB* in the Δ*sadB* mutant (Fig. 6C and D). To quantify the impact of deleting *sadB* on rhamnolipid production, LC-MS/MS was used. The Δ*sadB* mutant produced ~3.4- and ~9-fold more than the WT and *sadB^+^*, respectively (Fig. 6E). Genetic complementation of Δ*sadB* reduced rhamnolipid production by ~6-fold. In *sadB^+^,* rhamnolipid production was ~2.8-fold lower than in the WT (Fig. 6E).

Since rhamnolipids are biosurfactants that contribute to both biofilm maturation and swarming motility, their overproduction in Δ*sadB* could explain both the biofilm-defective and hyper-swarming phenotypes of this mutant. To explore this possibility, we deleted the *rhlA* gene in Δ*sadB*. Figure 6F and Fig. S3, S4E and F at https://doi.org/10.5281/zenodo.19057088 show that biofilm formation could be restored to WT levels in the Δ*sadB*Δ*rhlA* double mutant.

**Fig 7:**
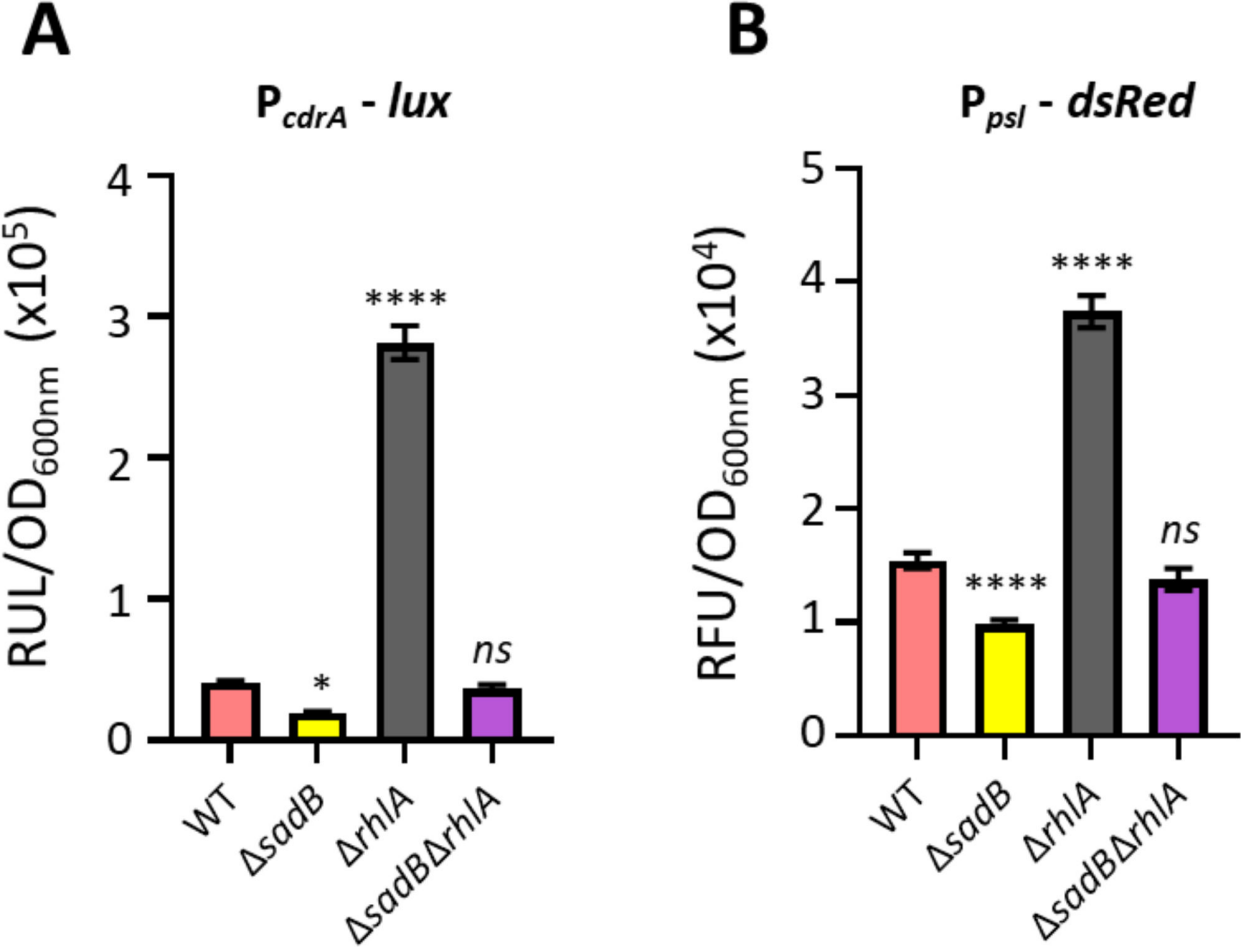
Deletion of the rhamnolipid biosynthesis gene, *rhlA,* results in increased expression of *cdrA* and *pslA,* which are reduced back to wild type levels in a Δ*sadB*Δ*rhlA* double mutant, respectively, reflecting changes in c-diGMP and the redirection of common biosynthetic precursors from rhamnolipid to Psl biosynthesis. (**A**) *cdrA’::lux* and (**B**) *pslA’::dsRed* promoter activity in Δ*sadB,* Δ*rhlA,* and Δ*sadB*Δ*rhlA* compared with wild type as a function of growth in LB at 37°C. Bars correspond to the area under the curve (AUC) derived from plotting relative light units normalized to culture density (RLU/OD_600_ or RFU/ OD_600_) over time. Errors represent SDs between three biological replicates. Statistical differences between group means were determined by one-way ANOVA using Tukey’s multiple comparisons test to the wild type (*P < 0.05, ****P < 0.0001).

Given the reciprocal regulatory links between exopolysaccharides and rhamnolipids via c-di-GMP signaling (35, 36), we investigated c-di-GMP levels indirectly using a *cdrA::lux* fusion (37) in a Δ*rhlA* mutant compared with Δ*sadB* and a Δ*sadB*Δ*rhlA* double mutant. Figure 7A shows that reporter expression was significantly elevated in Δ*rhlA* but reduced back to WT levels in Δ*sadB*Δ*rhlA,* consistent with the positive contribution of SadB to the c-di-GMP signaling network. To confirm these data, given that *cdr*A is also a target of AmrZ, we confirmed the results (Fig. 5E) using the alternative reporter described by Kaczmarczyk et al. (29). In further support of the role of SadB in biofilm formation, we observed that *pslA* expression was significantly upregulated in Δ*rhlA* but reduced back to WT levels in a Δ*sadB*Δ*rhlA* double mutant (Fig. 7B).

### SadB and quorum sensing

*P. aeruginosa* employs a quorum sensing cascade integrating two *N*-acyl (AHL) lactone systems (*las* and *rhl*) with an alkylquinolone (*pqs*) QS system (1). Since rhamnolipid biosynthesis is controlled via both the *rhl* and *pqs* QS systems (38), we explored the impact of SadB on QS in the RNA-seq data set. When compared with the WT and in common with the QS target genes, the *N*-butanoyl homoserine lactone (C4-HSL) synthase gene, *rhlI,* and the AQ biosynthesis genes (*pqsABCDE phnAB* and *pqsH*) were all highly upregulated in log-phase Δ*sadB* (see Table S2 at https://doi.org/10.5281/zenodo.19057088), consistent with a loss of population density dependency. Although the *lasRI* genes were not differentially regulated, the gene coding for RsaL, the homeostatic negative regulator of *las*-dependent QS (39), was downregulated in log-phase Δ*sadB*.

**Fig 8:**
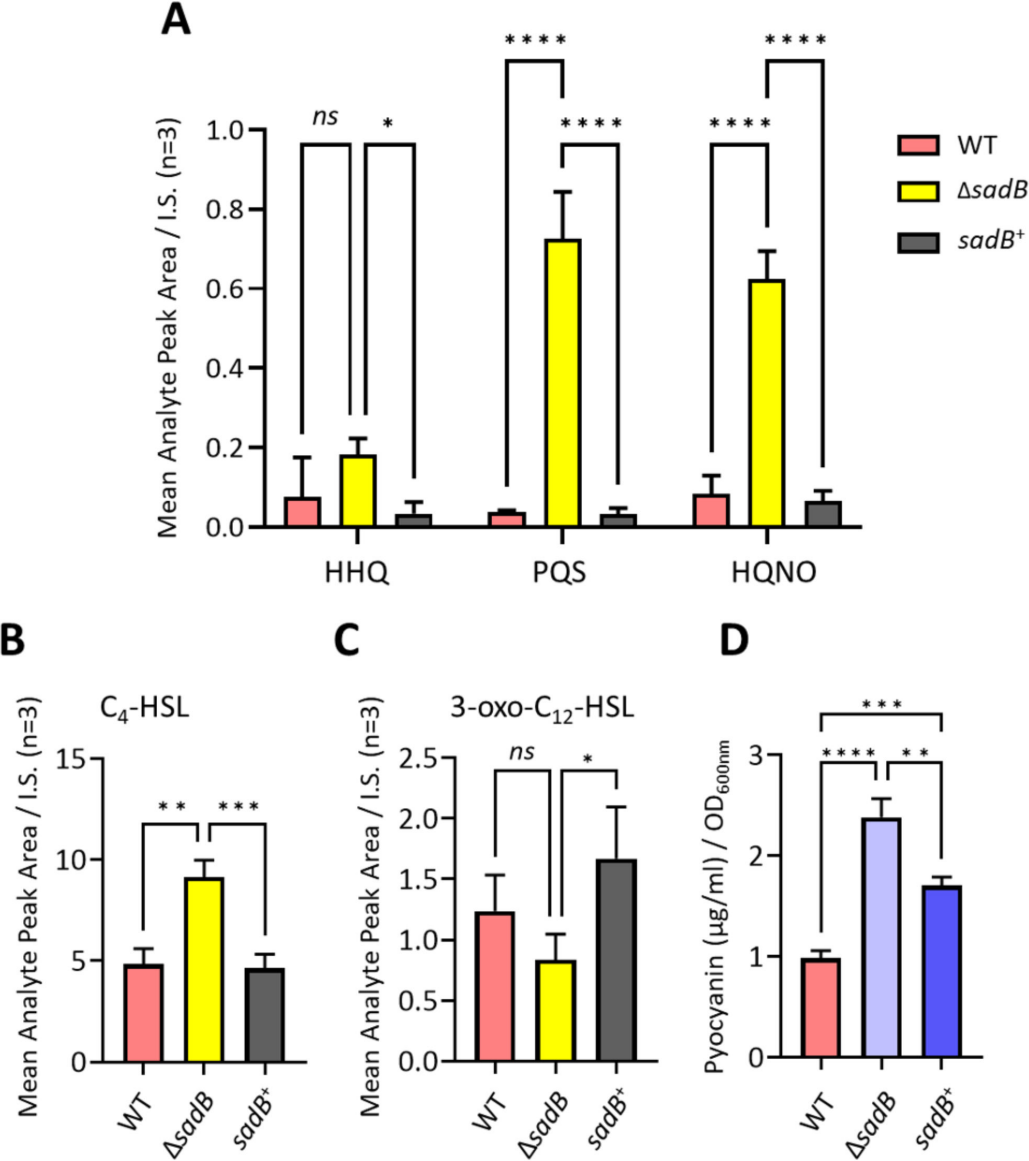
SadB positively regulates AQ and AHL signal molecule and pyocyanin production. (**A**) HHQ, PQS, and HQNO; (**B**) C_4_-HSL; (**C**) 3-oxo-C_12_-HSL; and (**D**) pyocyanin. AHLs and AQs in culture supernatants were quantified using LC-MS/MS, while pyocyanin was quantified spectrophotometrically. Bacteria were grown in LB as described for the RNA-seq experiments. Values represent the means of at least three biological replicates. Statistical significance was determined by one-way ANOVA using Tukey’s multiple comparisons test. (*P < 0.05, **P < 0.01, ***P < 0.005, ****P < 0.0001).

To validate the RNA-seq data for QS systems, we quantified the production of QS signal molecules in stationary-phase culture supernatants using LC-MS/MS. Figure 8 shows that, compared with WT and *sadB*^+^, deletion of *sadB* resulted in statistically significant elevated levels of the AQs, PQS, and 2-heptyl-4-hydroxyquinoline *N*-oxide (HQNO), and the RhlI-dependent, short-chain *N*-acylhomoserine lactone, *N*-butanoylhomoserine lactone (C4-HSL). However, the difference between WT and Δ*sadB* for the LasI autoinducer*, N*-(3-oxo-dodecanoyl)homoserine lactone (3-oxo-C12-HSL), was not significant (Fig. 8C).

AQ biosynthesis requires anthranilate which can be supplied via the anthranilate synthases TrpEG and PhnAB or by the degradation of tryptophan via the kynurenine (*kyn*) pathway (40). Table S2 at https://doi.org/10.5281/zenodo.19057088 shows that *phnAB* and *kynA,* but not *trpEG* expression, was higher in log-phase Δ*sadB* in log phase compared with the WT (*phnA* 17.9-fold, *phnB* 7.1-fold; *kynA* 2-fold). These data are consistent with the earlier requirement for anthranilate for AQ production in log-phase Δ*sadB*. Conversely, anthranilate can be redirected to the TCA cycle for energy through degradation via the *ant* and *cat* pathways (41). The expression of the *antABC* genes was significantly downregulated in stationary-phase Δ*sadB* (*antA* −16.5-fold, *antB* −9.5-fold, *antC* −5.6-fold) but highly upregulated in stationary-phase *sadB*^+^, as was the *cat* operon which is responsible for the degradation of the catechol product arising from AntABC-mediated enzymatic degradation of anthranilate (see Table S2 at https://doi.org/10.5281/zenodo.19057088). These data are consistent with the expression of *antR*, the positive transcriptional regulator for both the AntABC and CatABC degradation pathways, which was upregulated in the RNA-seq data for the *sadB*^+^ strain (see Table S2 at https://doi.org/10.5281/zenodo.19057088) and confirmed by RT-qPCR (see Fig. S5 at https://doi.org/10.5281/zenodo.19057088).

Given the higher log-phase induction of the *rhl* and *pqs* QS systems in Δ*sadB* compared with WT, we examined the RNA-seq data for genes known to be regulated via QS. Table S2 at https://doi.org/10.5281/zenodo.19057088 shows that apart from *rhlAB*, those required for the exoproteases (*lasB*, *lasA*, *aprA, and pepB* [PA2939]), pyocyanin (*phz1* and *phzB1*), hydrogen cyanide (*hcnABC*), and the MexGHI efflux pump were all upregulated in log-phase Δ*sadB*. In addition, the biosynthetic genes coding for the siderophores pyochelin (*pch*) and pyoverdine (*pvd*), known to be regulated via the PqsR-independent ferric ion chelating properties of PQS (42, 43), were also induced in log-phase Δ*sadB*.

To validate the relevant RNA-seq data, we examined the stationary-phase extracellular protein profile of Δ*sadB* by SDS-PAGE (see Fig. S6 at https://doi.org/10.5281/zenodo.19057088). The levels of two proteins identified by LC M/S as elastase (LasB) and the leucine aminopeptidase (PaAP) appeared to be present in greater abundance in Δ*sadB* compared with WT, consistent with the increased expression of the corresponding *lasB* (2.8-fold upregulated) and *pepB* (PA2939; 2.7-fold upregulated) genes, both of which are QS-regulated (44). The *rhl* and *pqs* QS-regulated blue-green phenazine pyocyanin is produced in *P. aeruginosa* via either of the two almost identical *phz1* and *phz2* operons together with *phzM* and *phzS* (45). RNA-seq analysis showed increased expression of *phz genes* in Δ*sadB* compared with the WT (*phzA1*, 33.2-fold, *phzB1* 8.7-fold) in exponential phase (see Table S2 at https://doi.org/10.5281/zenodo.19057088), whereas *phzA1* was downregulated in the *sadB^+^* in the stationary phase compared with Δ*sadB* (*phzA1*, 21-fold). Quantification of pyocyanin levels after growth in flasks with shaking confirmed the RNA-seq data, in that significantly more pyocyanin was produced by Δ*sadB* compared with both the WT and *sadB^+^* (Fig. 8D).

### *sadB* and denitrification

In microaerophilic or anaerobic environments, *P. aeruginosa* is capable of dissimilatory nitrate reduction for energy production that depends on four enzymatic complexes to reduce nitrate to nitrite (NarGHI), nitrite to nitric oxide (NirS), nitric oxide to nitrous oxide (NorCB), and, finally, nitrous oxide to dinitrogen (NosZ) (46, 47) In the log-phase Δ*sadB* RNA-seq data set, *nar*, *nir*, *nor,* and *nos* genes were the most downregulated (from −237 to −12.3-fold; see Table S2 at https://doi.org/10.5281/zenodo.19057088). In addition, nitrate reduction, and in particular the NarGHI enzyme complex, requires a molybdate co-factor generated via the *moaA1B1 moaEDC* gene products (48), which were also negatively regulated in log-phase Δ*sadB* (see Table S2 at https://doi.org/10.5281/zenodo.19057088). In the absence of nitrate, *P. aeruginosa* is able to utilize arginine as an energy source via the *arcDABC* operon that codes for the arginine deiminase pathway, which is inducible under oxygen limitation (49). In common with dissimilatory nitrate reduction, the *arcDABC* operon was substantially downregulated in log-phase Δ*sadB* (see Table S2 at https://doi.org/10.5281/zenodo.19057088).

**Fig 9:**
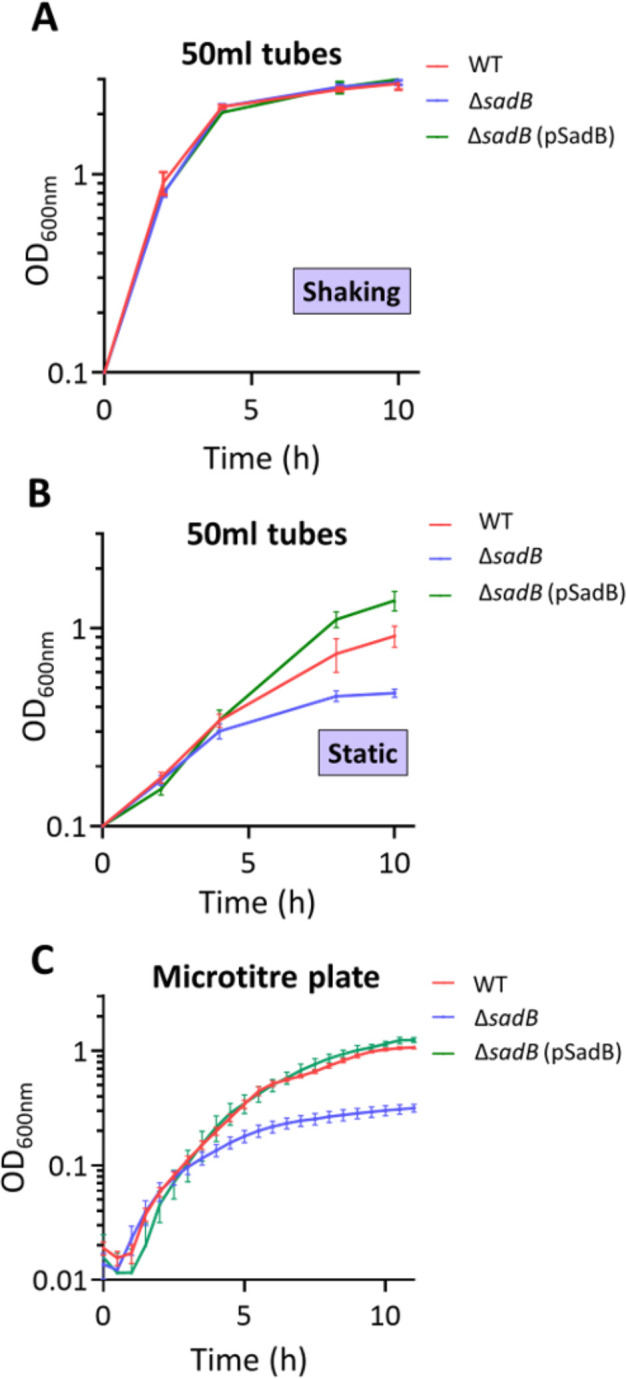
Oxygenation influences the growth of Δ*sadB*. Growth of WT and Δ*sadB* harboring the control vector pME6032Δ*lacIQ*, and Δ*sadB* pSadB in 50 mL conical tubes for 12 h in LB (5 mL) at 37°C under vigorous shaking (200 rpm) (**A**) or static (**B**) conditions, or (**C**) in a 96-well microtiter plate. Values given are averages from at least three different cultures ± standard deviation.

To evaluate the consequences arising from the reduced expression of genes required for growth under microaerophilic conditions, we grew the *P. aeruginosa* WT, Δ*sadB,* and *sadB*^+^ strains either statically or with shaking or in microtiter well plates. Under aerobic shaking conditions, all strains grew to similar cell densities (WT, OD_600_ 2.9 ± 0.1; Δ*sadB,* OD_600_ 2.9 ± 0.03; and *sadB^+^*, OD_600_ 3.0 ± 0.1) (Fig. 9A). In static conditions after 10 h of growth, there was a clear reduction in the Δ*sadB* growth (OD_600_ 0.47 ± 0.01) compared with the WT (OD_600_ 0.91 ± 0.04) or *sadB^+^* (OD_600_ 1.4 ± 0.1) (Fig. 9B).

In microtiter plates, the WT and complemented Δ*sadB* grown statically reached stationary phase and their maximum OD_600_ at ~10 h (Fig. 9C). A growth defect was clearly apparent for Δ*sadB,* in which growth began to slow between 3 and 4 h. Since downregulation of the denitrification genes in log-phase Δ*sadB* may be responsible for the microaerophilic growth defect, we supplemented LB with a range of nitrate concentrations. Figure S7 at https://doi.org/10.5281/zenodo.19057088 shows a small increase in Δ*sadB* growth under microaerophilic conditions when provided with 3.2 mM KNO_3_.

## DISCUSSION

The major aim of this work was to obtain new insights into the contribution of *sadB* to *P. aeruginosa* virulence and biofilm formation and to elucidate its downstream targets. In an acute mouse skin and soft tissue infection model, Δ*sadB* was unable to establish infection and was rapidly cleared from the infection site. This contrasted with both the WT parent and genetically complemented Δ*sadB,* both of which established within the host tissues, forming architecturally distinct infection sites. The higher luminescence output (~1.5 log fold) from the genetically complemented Δ*sadB* CTX::*tac’-sadB-luxCDABE* strain reflected an increased population of metabolically active bacteria on 3 and 4 days after inoculation. In addition, the greater size of the infection site, lack of a fibrotic pocket, and marked infiltration of leukocytes into both infection site and into the adipose and subdermal tissue layers further distinguished between WT and Δ*sadB* CTX::*tac’-sadB-luxCDABE* strain, indicating that the latter was more virulent. A PA14 *sadB* Tn insertion mutant has previously been reported to be attenuated in a rat model of chronic lung infection (50). These data are consistent with a broader role for SadB in the pathogenesis of *P. aeruginosa* infections than previously appreciated.

To evaluate the global impact of *sadB* deletion and to gain further insights into the reduction in virulence observed, we undertook RNA-seq analysis of planktonic cells, firstly by comparing WT with Δ*sadB* grown to either exponential or stationary growth phase. The data obtained surprisingly revealed that deletion of *sadB* resulted in the differential regulation of over 1,420 genes by ≥ ±2-fold in log-phase Δ*sadB* compared with WT. Far fewer genes were differentially regulated (~450 ≥ ±2-fold) by the time the cells reached stationary phase. Of particular interest was the log-phase induction of genes associated with the *P. aeruginosa rhl* and *pqs* QS systems in Δ*sadB,* indicating that SadB negatively influences QS via AmrZ (16, 18). Within the *pqs* system, PQS acts as an extracellular QS signal molecule and iron chelator that autoinduces AQ biosynthesis as well as the expression of genes involved in the iron-starvation response and virulence factor production via PqsR-dependent and PqsR-independent pathways (42). Given the impact of the *sadB* deletion on iron metabolism, it is noteworthy that impairment of iron release from bacterioferritin (BfrB), which is downregulated in log-phase Δ*sadB* (51), results in reduced biofilm formation but increased rhamnolipid production and swarming (51), that is, a similar phenotype to that of Δ*sadB*.

The dispensable *pqs* system thioesterase, PqsE, is involved in the regulation of diverse genes coding for key virulence determinants and biofilm development via a direct interaction with RhlR, the C4-HSL-dependent regulator (52). Although *rhlR* was not differentially regulated in log-phase Δ*sadB,* the C4-HSL synthase gene *rhlI* and the *rhlA* and *rhlB* genes required for rhamnolipid biosynthesis were all upregulated, consistent with the higher levels of C4-HSL and rhamnolipids quantified in stationary-phase Δ*sadB*. The log-phase induction of the *pqs* QS system is also in accordance with the increased expression of genes involved in high affinity iron transport systems, the downregulation of anthranilate degradation, and the substantially reduced expression of the *nir*, *nar,* and *nos* denitrification genes. The pivotal role of QS in the adaptation of *P. aeruginosa* to anaerobic growth is well established in the literature (53–55).

Since QS systems are, by definition, cell population density-dependent, their log-phase induction in Δ*sadB* may alert host defenses to the presence of low numbers of infecting bacteria and their virulence factors and so promote clearance before the pathogen becomes established. However, the log-phase induction of QS in Δ*sadB* is unlikely to provide a full explanation given that QS is just one of several different environmental parameters (e.g., temperature, pH, osmolarity, oxidative stress, nutrient deprivation) which bacterial cells must integrate to determine their optimal survival strategy within a given environment (56).

In microaerophilic but not aerobic conditions, Δ*sadB* exhibited a growth defect. Supplementation with nitrate, in contrast to the genetic complementation of *sadB,* only partially increased growth under microaerophilic conditions consistent with the downregulation of both the denitrification and molybdenum co-factor genes in Δ*sadB*. This could also conceivably compromise *P. aeruginosa* growth *in vivo,* where leucocyte-dependent utilization of oxygen to generate antibacterial free radicals in the tissues can cause localized hypoxia (57). In support of these observations, *sadB* has been reported in PAO1 to be expressed more highly under both low oxygen (58) and anaerobic conditions (59). These findings suggest that *sadB* is likely important for energy generation in oxygen-limiting conditions and for the normal operation of the denitrification pathway.

For both PA14 and PAO1, *sadB* mutants are hyper-swarmers that fail to form biofilms. The PA14 *sadB* mutant biofilm defect has been associated with an inability to switch from reversible attachment (where the bacterial cells are in a relatively unstable surface contact via their poles) to irreversible attachment where the cells are surface aligned via their long axes (10). Our finding that the rhamnolipid biosynthetic genes are upregulated in log-phase planktonic Δ*sadB,* leading to overproduction of rhamnolipids, suggested a further explanation of the Δ*sadB* biofilm defect. Rhamnolipids are multifunctional biosurfactants that have surface-lubricating, anti-adhesive properties and contribute to biofilm maturation and dispersal (60, 61). Premature overproduction of surface-lubricating rhamnolipids by Δ*sadB* is therefore likely to account for the inability of *P. aeruginosa* to switch from reversible to irreversible surface attachment. This suggestion is supported by our finding that deletion of *rhlA* in Δ*sadB* results in the restoration of biofilm formation with the double mutant forming a biofilm of similar biomass to the WT.

The RNA-seq data also revealed that deletion of *sadB* had a major impact on the transcription of many genes involved in c-di-GMP signaling. In PAO1, when c-di-GMP levels were maintained at low levels by constitutive expression of the PDE, YhJH, both *rhl* and *pqs* QS systems were expressed at higher levels (62), leading to increased production of *rhl* and *pqs*-regulated virulence factors including pyocyanin and rhamnolipids. This is consistent with the Δs*adB* data. However Lin Chua et al. (62), proposed that the induction of QS-regulated virulence factors in cells with low intracellular c-di-GMP levels and especially rhamnolipids would compromise host defenses and enhance virulence. Contrary to this, we found that Δ*sadB* was unable to establish infection and was readily cleared from the infection site in a mouse soft tissue infection model indicative of a less virulent phenotype that probably reflects the premature induction of QS and the pleiotropic impact of *sadB* on the transcriptome. Furthermore, the constitutive overexpression of *sadB* resulted in higher levels of c-di-GMP and a more persistent phenotype in our mouse infection model.

Apart from deleting *rhlA*, biofilm formation by Δ*sadB* could also be restored by expressing the DGC gene *siaD,* which was highly upregulated in log-phase *sadB*^+^. The *sia* system maintains low level c-di-GMP and Psl in planktonic *P. aeruginosa* such that *sia* mutants exhibit a surface attachment-deficient phenotype (30). Given the role of the *sia* operon in upregulating the production of Psl, which functions as an early stage adhesive, extracellular biofilm matrix component, and signal molecule (63), it is also possible that the restoration of biofilm formation in Δ*sadB* by SiaD is also a consequence of increased Psl production. This is possibly via c-di-GMP-dependent activation of Psl synthesis and also through diversion of biosynthetic substrates from rhamnolipid synthesis. Competition for common sugar precursors catalyzed via AlgC has been proposed as a mechanism for balancing the synthesis of Psl and rhamnolipids, so helping to coordinate the inverse control of swarming motility and biofilm formation (35, 36). Consistent with sugar precursor competition, we observed that both c-di-GMP levels and *psl* expression are significantly upregulated in a Δ*rhlA* mutant but reduced back to WT levels in the Δ*sadB*Δ*rhlA* double mutant, highlighting the importance of SadB in this context. Furthermore, the c-di-GMP PDE, NbdA, plays a key role in diverting substrates from Psl to rhamnolipids (35, 36). Interestingly, *nbdA* was highly upregulated in stationary-phase Δ*sadB* (see Table S2 at https://doi.org/10.5281/zenodo.19057088).

Although SadB from *P. fluorescens* F113 has been reported to function as a c-di-GMP binding protein (15), structural and c-di-GMP binding studies undertaken by Ben-David et al. (16) indicate that *P. aeruginosa* SadB, despite its similarity, is highly unlikely to require c-di-GMP or other small molecule ligands for activation. Instead, SadB was demonstrated to bind directly to the C-terminal domain of AmrZ, leading to the rapid proteolytic degradation of this transcription factor primarily via the Lon and to a much lesser extent by the AsrA and PepA proteases (16). Interestingly, we found that both *lon* and *asrA* genes were significantly upregulated in *sadB*^+^ stationary cells by 5.7- and 2.2-fold, respectively.

RNA-seq of log-phase PAO1 Δ*amrZ* compared with the genetically complemented mutant has revealed 338 differentially regulated genes (at least twofold; 89 up; 249 down) (18). Many of these genes are shared with the SadB regulon (1,421 twofold differentially regulated log-phase genes; Fig. 3B and C) including those involved in iron acquisition, c-di-GMP signaling, quorum sensing, biofilm formation, protein secretion, and motility. In the Δ*amrZ* RNA-seq data set (18)*, rhlA* was twofold downregulated whereas *rhlA* was highly upregulated in Δ*sadB* (this manuscript), consistent with the SadB-driven degradation of AmrZ and the respective swarming and biofilm phenotypes. It is therefore clear that while SadB clearly mediates substantial regulatory activity through AmrZ degradation, it may have additional targets given the apparently larger size of its regulon compared with that of AmrZ. Furthermore, the highly attenuated virulence of the *sadB* mutant, together with recent developments in the structure and function of SadB, suggests that it has considerable potential as a novel protein target for structure-based antibacterial drug discovery.

## MATERIALS AND METHODS

### Bacterial strains, plasmids, oligonucleotides, and culture conditions

The bacterial strains, plasmids, and oligonucleotides used in this study are listed in Tables S1 and S3 at https://doi.org/10.5281/zenodo.19057088. *P. aeruginosa* and *E. coli* strains were routinely cultured in LB at 37°C with shaking at 200 rpm or on LB agar. For some experiments, bacteria were also grown statically in LB with or without a range of potassium nitrate concentrations. Where required, the following antibiotics were added: ampicillin (Ap) 100 µg/mL (*E. coli*); carbenicillin (300 μg/mL) (*P. aeruginosa*); tetracycline (Tc) 25 µg/mL (*E. coli*) or 125 µg/mL (*P. aeruginosa*) and nalidixic acid (NA) 30 μg/mL (for *P. aeruginosa* selection in mating experiments with *E. coli*). *P. aeruginosa* swarming assays were carried out in Petri dishes on a solid medium containing Nutrient Broth No. 2 (Oxoid) 8 g/L, D-glucose 0.5% wt/vol, and Bacto agar (0.5% wt/vol; Difco) as described by reference Rampioni et al. (38). Congo red assay was adapted from previously published protocols (64). Briefly, LB agar (1%) plates supplemented with Congo Red (40 µg. mL^−1^) and Coomassie brilliant blue (20 µg. mL^−1^) were spotted with 2 μL of an overnight culture and incubated at 37°C for 16 h, followed by a further 4 days at room temperature.

### Construction of *P. aeruginosa* deletion mutants and complementation vectors

*P. aeruginosa* Δ*sadB* and Δ*sadB*Δ*rhlA* deletion mutants were constructed via allelic exchange. Two PCR products amplifying the upstream and the downstream regions of each gene were generated using the primer pairs 1FW/1RW and 2FW/2RW, respectively (see Table S3 at https://doi.org/10.5281/zenodo.19057088). The resulting PCR products were cloned into the suicide plasmid pME3087 (65) and introduced into *P. aeruginosa* via conjugation with *E. coli* S17-1 λpir. Recombinants were selected on tetracycline, followed by enrichment with carbenicillin (66). Deletions were confirmed by DNA sequence analysis, and their swarming and biofilm phenotypes were confirmed (Fig. 1). The *sadB* and *siaD* expression vectors (pSadB and pSiaD; see Table S1 at https://doi.org/10.5281/zenodo.19057088) were constructed by PCR amplification using PAO1 chromosomal DNA as a template and with primer pairs SadB F/R and SiaD F/R, respectively. *sadB or siaD* were each cloned into the shuttle vector pME6032, which contains a *lacIQ* mutation rendering the P*tac* promoter constitutive (67). The *sadB* gene was also cloned into the pCold-1 protein expression vector to generate pSadBcold (see Table S1 at https://doi.org/10.5281/zenodo.19057088) and transformed into *E. coli* BL21. After culturing in Terrific Broth, *sadB* expression was induced with IPTG (1 mM) after cold shock, and the bacterial cells were harvested by centrifugation and lysed. The lysate was subjected to nickel chromatography, followed by size-exclusion chromatography to purify the His-tagged SadB protein. Polyclonal antibodies against SadB were raised in rabbits.

### Construction of bioluminescent strains for *in vivo* experiments

The miniCTX::*tac’-luxCDABE* promoter fusion described previously (24) was introduced onto the chromosomal CTX site of both the WT parent and Δ*sadB*. In addition, a CTX::*tac’-sadB-luxCDABE* expression vector was constructed by PCR amplification of the *sadB* gene using PAO1 chromosomal DNA as a template and the primer pairs P_tac_sadBlux F/R (see Table S3 at https://doi.org/10.5281/zenodo.19057088). The resulting PCR product carrying the *sadB* gene was ligated into the miniCTX*lux* vector (see Table S1 at https://doi.org/10.5281/zenodo.19057088) using the appropriate restriction enzyme to generate miniCTX::*tac’-sadB-luxCDABE*. The resulting *lux*-marked *sadB* expression vector was integrated into the chromosome of *P. aeruginosa* by conjugation with *E. coli* S17.1 λpir, followed by selection with tetracycline.

### Construction of *pslA’*::*lux* transcriptional reporter fusion

The promoter regions of *pslA* were amplified from the PAO1 genome using the primer pair PpslA’F/R (see Table S3 at https://doi.org/10.5281/zenodo.19057088). The resulting PCR product was ligated into the mini-CTX*lux* vector using the appropriate restriction enzyme to generate the mini-CTX::*pslA’-lux* transcriptional reporter. The transcriptional reporter was integrated into the *P. aeruginosa* chromosome by conjugation with *E. coli* S17.1 λpir, followed by selection with gentamicin.

### Mouse infection model and *in vivo* imaging

A simple and reproducible mouse model based on that described by Bunce et al. (20) was used. Female BALB/c mice, 19 g–22g were housed in individually vented cages under a 12 h light cycle, with food and water *ad libitum*. Animals were anaesthetized with 2% isoflurane, their flanks shaved, and the skin cleaned with Hydrex surgical scrub. *P. aeruginosa* WT and Δ*sadB* strains carrying chromosomally integrated CTX::*tac’-luxCDABE* or CTX::*tac’-sadB-luxCDABE* fusions (1 × 10^5^ cfu; see Table S1 at https://doi.org/10.5281/zenodo.19057088) were mixed with Cytodex 1 microcarrier beads (20), 1 μg/50 μL total volume phosphate-buffered saline pH 7.4, and co-injected subcutaneously. Animals (four mice per experimental group) were allowed to recover and monitored throughout the 4 day study. The progress of bacterial infection was tracked non-invasively by following bioluminescence output using an IVIS Spectrum (PerkinElmer) imager (22, 23). Infected animals were imaged daily for 4 days for the presence of bioluminescent bacteria at the infection sites. Mice were humanely euthanized, and infection site tissues excised for histological assessment were dissected, fixed, and stained with hematoxylin and eosin as described by Singh et al. (23). Bacterial cells were visualized by IHC using polyclonal antibodies raised against *P. aeruginosa* (Invitrogen PA1-73116) and detected with an anti-rabbit Alexa 555 antibody conjugate (Thermo Fisher). Tissues were counterstained for DNA with POPO-1. Slides were mounted with Fluoromount (Sigma-Aldrich). Images were acquired on a Zeiss Axioscan 7 slide scanner, objective 20×.

### RNA extraction, cDNA preparation, and RT-qPCR

Bacteria (WT, Δ*sadB,* and *sadB*^+^) were grown in 5 mL of LB in 50 mL conical tubes at 37°C with shaking at 200 rpm and harvested at early exponential phase (OD_600_ 0.7) or stationary phase (OD_600_ ~2.0; 19 h). A representative growth curve is shown in Fig. 9A. RNA was extracted using the RNeasy Mini Kit (QIAGEN) together with RNAprotect Cell Reagent (QIAGEN) to preserve RNA integrity. Residual genomic DNA was digested using a Turbo DNA-free kit (Invitrogen) and PCR to confirm the absence of DNA contamination. RNA quality and quantity were assessed using an Agilent 2100 Bioanalyzer and the Agilent RNA 6000 Nano Kit. The RNA obtained was used as a template for cDNA synthesis using the GoScript Reverse Transcriptase kit (Promega, USA). qPCR for the relative expression of target transcripts was performed using an ABI 7500 Fast Real-Time PCR System (Applied Biosystems) and Power SYBR Green PCR Master Mix according to the manufacturer’s instructions. The oligonucleotides used for qPCR are listed in Table S3 at https://doi.org/10.5281/zenodo.19057088. The *rpoS* gene was used as the internal “housekeeping” control to normalize the qPCR data, as it exhibited the least variation in expression in the RNA-seq data set. The analysis was performed at least in duplicate on three technical replicates.

### Transcriptome analysis

RNA sequencing and data analysis were carried out by Novogene. Sequencing libraries were generated using the NEBNext Ultra RNA Library Prep Kit for Illumina (NEB, USA). The RNA-seq results have been deposited in NCBI’s Gene Expression Omnibus and are accessible through GEO Series accession number GSE302429 (https://www.ncbi.nlm.nih.gov/geo/).

### Biofilm formation and imaging

Biofilms were grown on borosilicate glass coverslips in RPMI-1460 (Lonza) at a temperature of 37°C for a period of 24 h. Biofilms were stained with FM4-64 dye (5 μM) and were visualized using a confocal laser scanning microscope (LSM700, Carl Zeiss) and 555 nm laser. At least five replicate Z-stack biofilm images were taken randomly using Zen 2011 imaging software. Biomass was quantified using ImageJ (NIH, Bethesda, MD, USA) and Comstat 2.1 (https://www.comstat.dk/) (68).

### Bioluminescence and fluorescence reporter assays

To evaluate the expression of *cdrA’::lux* and *pslA’::lux* and CdGreen2.1 (see Table S1 at https://doi.org/10.5281/zenodo.19057088) reporter gene fusions as a function of growth, bacteria were grown in 96-well plates (flat black transparent bottom, Greiner Bio-One). OD_600_ and luminescence (RLU) or fluorescence (RFU) measurements were taken every 30 min at 37°C for 24 h using the TECAN Infinite F200Pro device. Each assay was performed independently at least twice with three technical replicates.

### QS signal molecules, pyocyanin, and rhamnolipid quantification

For QS signal molecule and pyocyanin quantification, the bacteria were grown in LB under the same conditions used for the RNA-seq. The AHLs, C4-HSL and 3-OC12-HSL, and the AQs (PQS, HHQ, and HQNO) were quantified using LC-MS/MS after extraction from culture supernatants with acidified ethyl acetate as described by Ortori et al. (69). Pyocyanin was extracted with chloroform prior to LC-MS/MS. Rhamnolipids were quantified by LC-MS/MS after growth to support maximum production in M9 medium containing 2% wt/vol glycerol, 2 mM MgSO_4_, and 0.05% wt/vol glutamic acid at 37°C with shaking at 200 rpm (70).

### Siderophore assays

Total siderophores present in spent cell-free culture supernatants prepared from *P. aeruginosa* were quantified using the CAS assay (71) with desferrioxamine as a positive control. Since maximum siderophore production and quantification requires an iron-deficient medium that does not interfere with the CAS assay, for these experiments we grew *P. aeruginosa* in casamino acids (CAA) medium (72). Pyoverdine was quantified spectrophotometrically as described by Diggle et al.(43) by determining the absorbance of supernatants at 405 nm (A_405_) and dividing by OD_600_.

### Extracellular protein profiles

Cell-free culture supernatants from *P. aeruginosa* WT, Δ*sadB,* and *sadB*^+^ grown to stationary phase with shaking in LB at 37°C (as described for the RNA-seq experiments) were treated with trichloroacetic acid (10% vol/vol) for 30 min on ice. The precipitated proteins were collected by centrifugation, washed with acetone prior, and air-dried. Samples were heated at 90°C for 5 min in sample buffer prior to SDS-PAGE. After staining with Coomassie blue, selected bands were excised and sent for MALDI-MS/MS. Experiments were repeated at least three times.

### SDS-PAGE and Western blotting

*P. aeruginosa* cytoplasmic fractions were prepared from WT, Δ*sadB,* and *sadB*^+^ grown to stationary phase with shaking in LB at 37°C (as described for the RNA-seq experiments). Cultures were normalized to the same OD_600_, and the cells were harvested by centrifugation. Bacteria were treated with lysozyme (100 μg/mL) for 1 h prior to lysis in a FastPrep-24 system. Cell membranes were removed by centrifugation, and the cytoplasmic fraction was subjected to SDS-PAGE and transferred to a nitrocellulose membrane. After blocking with Tris-buffered saline (pH 8) containing 5% wt/vol skimmed milk powder, the nitrocellulose was probed with a polyclonal rabbit antibody raised against the purified SadB protein, followed by HRP-conjugated anti-rabbit IgG, and the blots were exposed to Hyperfilm (GE) after incubation with an enhanced chemiluminescence HRP substrate (Thermo Fisher).

### Statistical analysis

One-way and two-way ANOVA analyses using Tukey’s multiple comparison tests were applied to determine whether *P. aeruginosa* derivative strains’ responses differed significantly from that of the parental strain (*P* < 0.05) when compared with the variations within the replicates (*n* ≥ 3) using GraphPad Prism 8.0 (GraphPad Software, Inc., San Diego, CA).

## Data Availability

The RNA-seq results have been deposited in NCBI’s Gene Expression Omnibus and are accessible through GEO Series accession number GSE302429. Supplemental material is available for download from Zenodo (https://zenodo.org/records/19057088; doi: 10.5281/zenodo.19057088).
